# Fatigue life of additively manufactured Ti6Al4V scaffolds under tension-tension, tension-compression and compression-compression fatigue load

**DOI:** 10.1038/s41598-018-23414-2

**Published:** 2018-03-21

**Authors:** Karel Lietaert, Antonio Cutolo, Dries Boey, Brecht Van Hooreweder

**Affiliations:** 1 3D Systems - LayerWise NV, Grauwmeer 14, Leuven, Belgium; 2 KU Leuven Department of Materials Engineering, Kasteelpark Arenberg 44, Leuven, Belgium; 3 KU Leuven Department of Mechanical Engineering, Celestijnenlaan 300, Leuven, Belgium

## Abstract

Mechanical performance of additively manufactured (AM) Ti6Al4V scaffolds has mostly been studied in uniaxial compression. However, in real-life applications, more complex load conditions occur. To address this, a novel sample geometry was designed, tested and analyzed in this work. The new scaffold geometry, with porosity gradient between the solid ends and scaffold middle, was successfully used for quasi-static tension, tension-tension (R = 0.1), tension-compression (R = −1) and compression-compression (R = 10) fatigue tests. Results show that global loading in tension-tension leads to a decreased fatigue performance compared to global loading in compression-compression. This difference in fatigue life can be understood fairly well by approximating the local tensile stress amplitudes in the struts near the nodes. Local stress based Haigh diagrams were constructed to provide more insight in the fatigue behavior. When fatigue life is interpreted in terms of local stresses, the behavior of single struts is shown to be qualitatively the same as bulk Ti6Al4V. Compression-compression and tension-tension fatigue regimes lead to a shorter fatigue life than fully reversed loading due to the presence of a mean local tensile stress. Fractographic analysis showed that most fracture sites were located close to the nodes, where the highest tensile stresses are located.

## Introduction

Ti6Al4V is the most popular material for implant production today because of its high strength, high corrosion resistance and good biocompatibility^1^. As the material is difficult and thus expensive to machine, Additive Manufacturing (AM) of Ti6Al4V has gained a lot of interest in the last years^2^. Since most Ti6Al4V implants have a structural function and an intended lifetime of several decades, their fatigue performance is of vital importance. The fatigue of AM Ti6Al4V has been studied by different research groups and includes both Selective Laser Melted (SLM) and Electron Beam Melted (EBM) material^3^. Its fatigue performance is, just like conventionally processed material, governed by surface roughness, residual stress, manufacturing defects, microstructure, and loading conditions^3^. If properly processed, from raw material through AM to heat treatment and surface treatments, AM Ti6Al4V can outperform conventionally produced materials because of its finer microstructure^3^.

Additive manufacturing allows seamless integration of porous volumes in implants, which lowers the stiffness of the implant and reduces stress shielding^4^. In addition, the open pore space allows bone ingrowth and can be functionalized to accelerate bone regeneration^5,6^. The design freedom offered by AM enables the production of scaffolds with controlled unit cell architectures, which can be optimized for fatigue performance, impact resistance, etc. However, the fatigue properties of Ti6Al4V scaffolds are usually lower than expected from theory^7^. Some of the fatigue-influencing aspects mentioned above are more important for scaffolds than for non-cellular material. First, their high surface area to volume ratio increases the influence of the high AM surface roughness and leads to early crack initiation^8^. Second, scaffolds are more difficult to produce than non-cellular parts and thus contain relatively many defects. This is caused by the small length of the scan tracks used to build scaffolds, which increases the influence of the non-steady-state melting regime at the beginning and end of every track^9–11^. Defects in struts are always located close to a free surface and this position strengthens their negative influence on fatigue life^12,13^. In addition, due to other manufacturing constraints (e.g. dross formation on down-facing surfaces) the architecture of the as-manufactured scaffolds deviates from the ideal, which can lower mechanical performance^14^.

Mechanical properties of AM Ti6Al4V scaffolds, both quasi-static and dynamic, have mainly been studied in uniaxial compression^7,15–24^. However, implants are subjected to more complicated load conditions over their lifetime and different fatigue regimes can occur. AM Ti6Al4V scaffolds have been tested in quasi-static tension and cyclic tension-compression loading in a limited number of studies^25,26^. Although valuable insights in tension-compression fatigue behavior of scaffolds with cubic unit cells were obtained, scaffolds were never tested in tension-tension before and no S-N curves were published so far^26^. Therefore, the aim of this work was to make a detailed study of the mechanical properties of SLM based Ti6Al4V ELI scaffolds with diamond unit cells in compression-compression, tension-tension and tension-compression fatigue for different stress amplitudes. For this purpose, a novel sample geometry with gradient porosity was designed and successfully tested in fatigue, which resulted in the construction of an S-N curve for every load condition. Experimental data was analyzed by applying local stress based fatigue analysis, which resulted in new insights in the failure of SLM based Ti6Al4V scaffolds under clinically and industrially relevant load conditions.

## Materials and Methods

**Figure 1:**
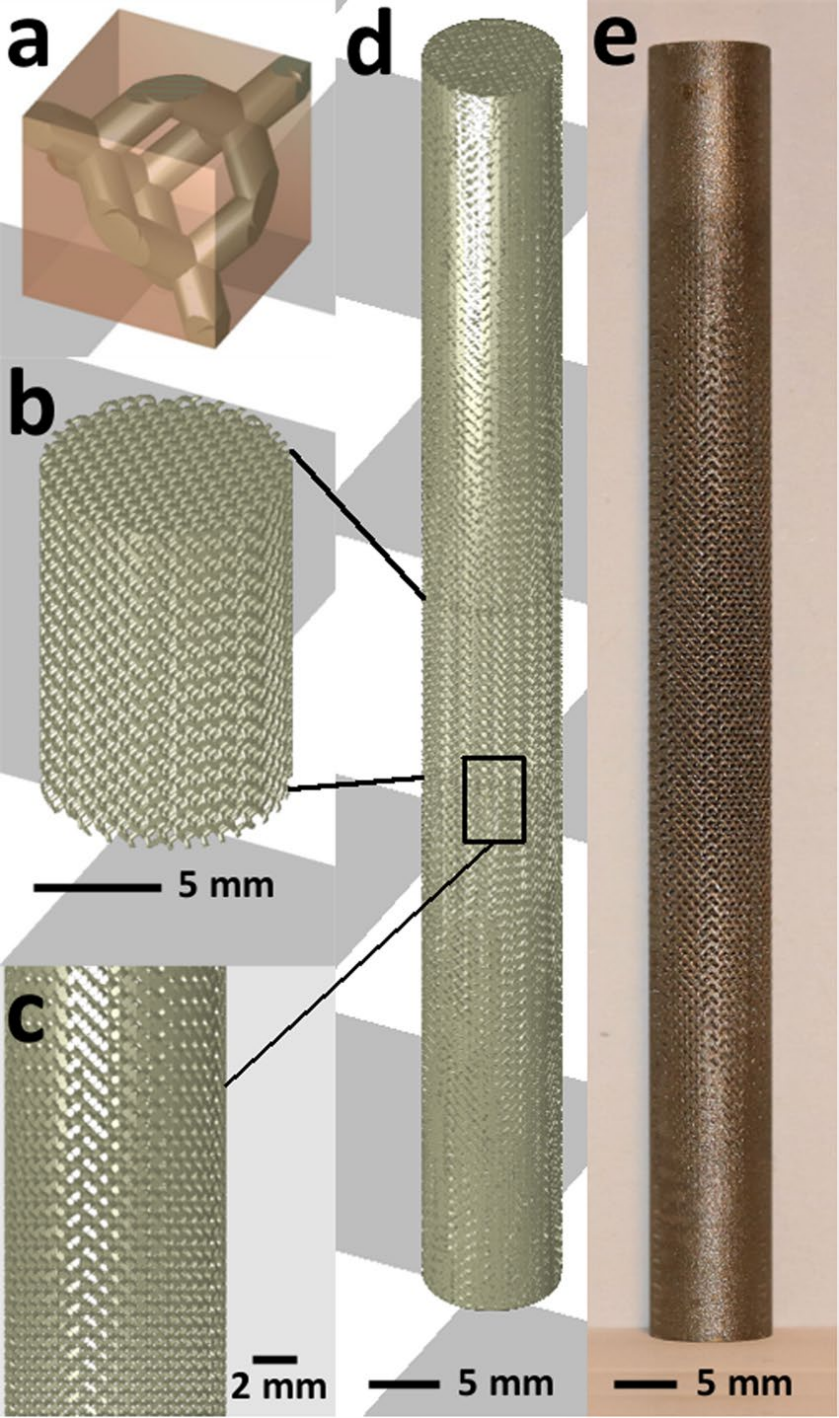
Sample design: (**a**) Diamond unit cell, cell size 1 mm, used to build up the samples. (**b**) The central part of the samples used for mechanical testing had a designed structural density of 20%, a diameter of 10 mm and a height of 15 mm. (**c**) In order to clamp the samples during testing, the structural density of the samples increased linearly towards the end (20% to 100%). (**d**,**e**) The full sample used for mechanical testing had a height of 105 mm.

The 3DXPert software package and DMP Control software (3D Systems) were used for sample design, slicing and hatching. A diamond unit cell with cell size 1 mm was used, Fig. 1a. The struts of this unit cell are oriented favorably for DMP: none of the struts has its longitudinal axis parallel to the horizontal plane and therefore the amount of dross formation is reduced. More details on sample design can be found in previously published work^23^. The middle of the sample had a constant designed structural density of 20% and a height of 15 mm, Fig. 1b, and conforms to the ISO 13314 standard^27^. In order to clamp the samples during testing, the structural density of the samples increased linearly towards the end (20% to 100%), Fig. 1c–e. The total height of the samples was 105 mm and their diameter 10 mm. A total of 60 samples was produced and used for all mechanical tests. In addition, seven samples which consisted of only the central part, Fig. 1b, were used to measure the as-produced structural density of the samples and determine possible manufacturing inaccuracies based on a Computed Tomography (CT) scan.

Samples were produced on a ProX DMP320 machine with LaserForm Ti Gr23 powder (3D Systems) which conformed to the ASTM F3001 standard^28^. The powder layer thickness was 30 µm and the standard production parameters from 3D Systems for this material were used. The samples were built on a pure Ti baseplate and removed from this plate by electrical discharge machining. The powder in the pores was removed by ultrasonic cleaning in demineralized water. No heat treatment or surface treatment was performed.

For seven scaffolds with geometry of Fig. 1b the structural density was measured. Their mass was measured with a Sartorius Acculab balance with 0.1 mg resolution. Their volume was calculated from height and diameter, measured with a digital caliper. These were measured 3 times per sample and averaged to calculate the volume. The dry mass and mass submerged in ethanol were used to calculate strut density from the Archimedes principle. One of these samples was also analyzed with a Nikon XT H 225 ST CT-system to assess the difference in geometry between the designed and printed unit cells. A W target and 1 mm Cu filter were used during scanning and the machine was set to a voltage of 160 kV and current of 120 µA. The voxel size was 14.5 µm. Data analysis and stl reconstruction was performed with VGSTUDIO software (VolumeGraphics). The CT-based stl and an stl with diamond unit cell and relative density as measured on the produced samples were compared in CloudCompare open-source software.

Tensile testing was performed on an Instron 4505 machine with 100 kN load cell and a clip-on extensometer. The samples were clamped over a length of 30 mm at both extremities. The central 12.5 mm of the sample was used as gauge length. Three samples were tested with a displacement rate of 0.9 mm/min. The slope of the initial linear part of the stress-strain curve was used as a measure of stiffness. This was offset by 0.2% strain to find the yield strength. The ultimate tensile strength and elongation at break were calculated as well.

Low frequency cyclic tests to measure possible asymmetry in tensile and compressive behavior were performed on an Instron Electropuls E10000. Two samples were again clamped over a length of 30 mm at both extremities and were loaded with a piecewise linear triangular wave force with mean load of 0 N for five complete cycles (0.9 mm/min displacement rate). The strain was measured by an Instron Video Extensometer AVE2. The tests were performed for three stress amplitudes: 10, 30 and 50 MPa.

Force-controlled fatigue tests with constant amplitude sinusoidal load (15 Hz) were performed on an Instron Electropuls E10000. A stress ratio of 10 was used for the compression-compression tests, a stress ratio of 0.1 for tension-tension tests and a stress ratio of −1 for tension-compression tests. Table 1 shows the different stress amplitudes (*σ*_*a*_) used for every stress ratio (R). As damage accumulates, the stiffness of the scaffold decreases. The stiffness can therefore be used as a criterion for failure and the tests were stopped if the elastic stiffness compared to the 500^th^ cycle decreased by 55% or if the sample failed completely. If neither of these criteria was met after 10^6^ cycles, the test was stopped as well (run-out). At least 2 samples were tested for every stress amplitude. S-N curves were constructed by plotting the fatigue life for every stress value. All samples fractured in the section with constant porosity, the results can thus be considered true scaffold properties.

**Table 1:** Different stress amplitudes used to construct S-N curves for different stress ratios (R).

Load regime	R	*σ*_*a*_,_1_	*σ*_*a*_,_2_	*σ*_*a*_,_3_	*σ*_*a*_,_4_	*σ*_*a*_,_5_	*σ*_*a*_,_6_
		MPa	MPa	MPa	MPa	MPa	MPa
Compression-compression	10	9	11.25	13.5	18	22.5	
Tension-tension	0.1	6.75	9	11.25	13.5	15.75	18
Tension-compression	−1	7	10	15	20	25	

**Figure 2:**
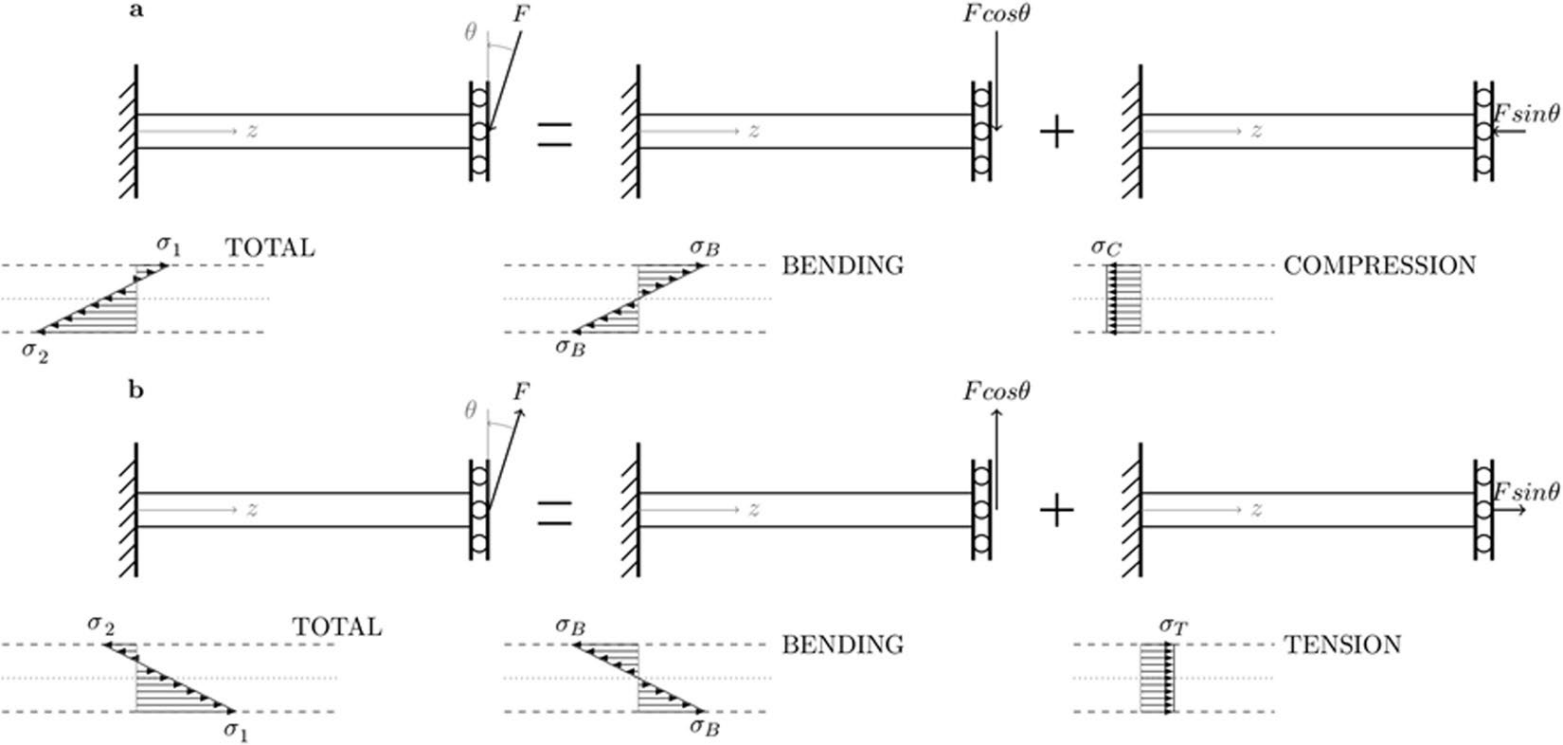
An approximation of the normal stresses in a hyperstatic strut: (**a**) A strut in a scaffold loaded in (global) compression experiences a small maximum local tensile and a large maximum local compressive stress. (**b**) A strut in a scaffold loaded in (global) tension experiences a large maximum local tensile and a small maximum local compressive stress.

The stresses in Table 1 were calculated by dividing the force by the circular cross-section of the sample and are referred to as ‘global’ stresses in this study. Global stress is contrasted with ‘local’ stress, which is an approximation of the stress acting on the struts^23^. When a scaffold with diamond unit cells is loaded uniaxially, the local stress σ in a strut consists of a uniform axial stress (*σ*_*C*_ or *σ*_*T*_) and a bending stress gradient (*σ*_*B*_), Fig. 2. Crack initiation and propagation are mostly caused by tensile stresses, therefore they can be considered the most important driver for strut failure. For global compression, the calculation of the maximum local tensile stress *σ*_1_ is based on Fig. 2a, Equation (1)^23^. In this equation *F* is the local force, *L* the strut length, *d* the strut diameter and *θ* the angle between the strut end and the load direction^23^. For the scaffolds used here, *L* is around 430 µm, d around 220 µm, and *θ* equal to 35.26°^23,29^. For a scaffold loaded in tension, the stress distribution is shown by Fig. 2b. In this case the maximum local tensile stress *σ*_1_, Equation (2), is larger than for the compressive case. Based on these equations, local tensile stress amplitudes can be evaluated and local S-N curves can be defined. These local stress amplitudes are evaluated in the region of the struts where the maximum local tensile stress *σ*_1_ occurs: at the surface in proximity of the nodes. This model considerably simplifies the stress distribution in the scaffold and fails to incorporate e.g. less constrained struts on the outer surface of the scaffold, geometrical non-linearities, stress concentrations, surface roughness, residual stresses, internal pores… Despite these limitations, the model was found to be useful in compression-compression fatigue and is therefore used in this research as well^23^.

1 $${\sigma }_{1}=F\,(\frac{16Lcos\theta }{\pi {d}^{3}}-\frac{4sin\theta }{\pi {d}^{2}})=\frac{4F}{\pi {d}^{2}}(\frac{4Lcos\theta }{d}-sin\theta )=\frac{4F}{\pi {d}^{2}}(6.4-0.6)$$

2 $${\sigma }_{1}=F\,(\frac{16Lcos\theta }{\pi {d}^{3}}+\frac{4sin\theta }{\pi {d}^{2}})=\frac{4F}{\pi {d}^{2}}(\frac{4Lcos\theta }{d}+sin\theta )=\frac{4F}{\pi {d}^{2}}(6.4+0.6)$$

The fracture surface after quasi-static tensile and fatigue testing was observed with an XL30 Scanning Electron Microscope (SEM, FEI) in secondary electron mode.

All measures of variation given in this study are standard deviations.

## Results and Discussion

### Morphological characterization

The structural density of the scaffold was on average equal to 29.81 ± 0.44% and the strut porosity was on average equal to 1.25 ± 0.11%.

**Figure 3:**
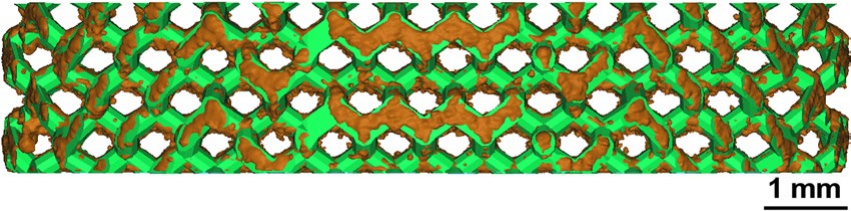
The manufactured scaffold (brown) has a much higher surface roughness than the designed scaffold (green), especially on the downfacing surface of the struts. Despite this difference, the designed scaffold is properly replicated by the manufactured geometry.

Despite a generally high surface roughness, in particular on the downfacing surface of the struts, the as-printed geometry properly replicates the designed geometry without affecting the bending-dominated mechanical behavior of the diamond unit cell, Fig. 3. Therefore, no change in deformation or failure mechanism is introduced by manufacturing inaccuracies. A quantitative analysis of the difference between designed and manufactured geometry could support this, but was not performed here^30^.

### Quasi-static tests

**Figure 4:**
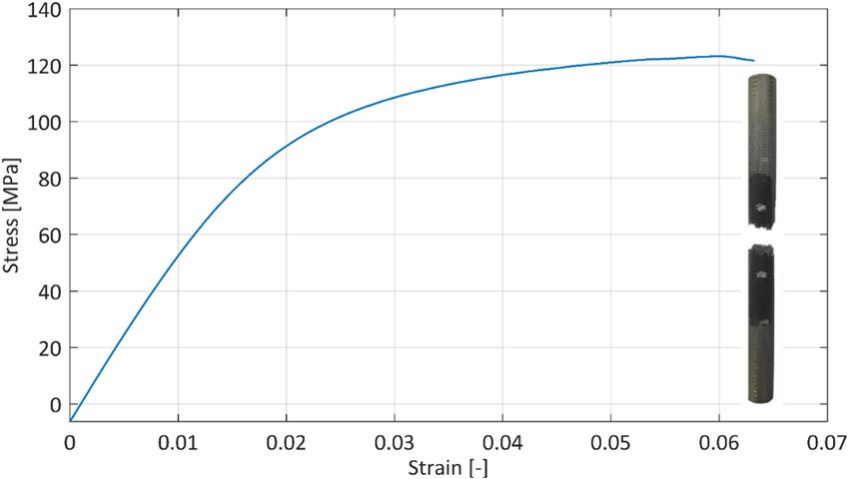
Samples deform in a ductile manner in a quasi-static tensile test and show an average yield stress of 70.18 MPa and an average fracture strain of 6.70%. The inset shows the macroscopic fracture behavior in quasi-static tension.

During a tensile test the scaffolds initially showed a linear relation between stress and strain and then strain hardened until sudden fracture, Fig. 4. Their stiffness was 5.84 ± 0.20 GPa, yield stress 70.18 ± 5.95 MPa, ultimate tensile stress 123.46 ± 1.50 MPa and fracture strain 6.70 ± 0.38%. The fracture surface was oriented quasi-perpendicular to the load direction, Fig. 4. This behavior was also observed in tensile testing of scaffolds with cubic unit cells^26^. The samples tested in the present work are stiffer than those studied in^26^ but due to the different unit cell and difference in porosity, absolute values are difficult to interpret. Compared to the results for non heat-treated samples described in^25^, two differences stand out. First, the curve shown in Fig. 4 lacks the stress drops reported in^25^. Second, the fracture surface is oriented differently in this study compared to^25^: Brenne *et al*. report a 45° orientation difference between the fracture surface and the loading direction. Given the as-manufactured microstructure in both^25^ and this work, the different tensile behavior should be related to differences in sample design (macroscopic) and/or internal structure (mesostructure). For example, the use of a density gradient from the extremities to the center of the sample in this study changes the stress distribution compared to^25^. Other important factors are the amount of unit cells in the smallest sample dimension, the unit cell geometry and deviations from the designed geometry^14,31–33^.

**Figure 5:**
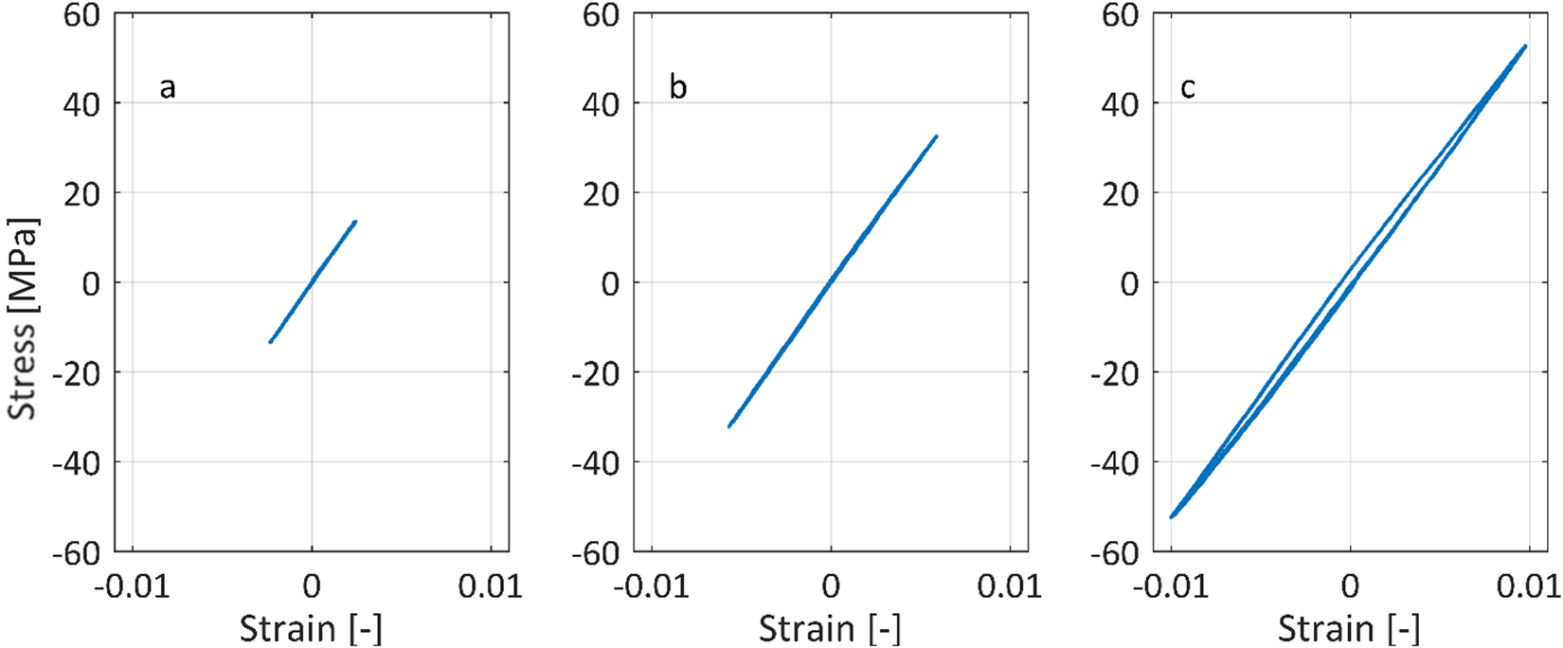
Stress-strain diagram for low frequency cyclic tests performed with different stress amplitudes around 0 MPa mean stress: (**a**) 10 MPa stress amplitude. (**b**) 30 MPa stress amplitude. (**c**) 50 MPa stress amplitude.

The results of the low frequency cyclic tests show that the samples behave similarly in tension and compression up to a global stress of 50 MPa, Fig. 5. Given the symmetry of the diamond unit cell and sample, this result could be expected for stresses below the yield stress. As no global stresses higher than 50 MPa were used in the fatigue tests, similar behavior during the tensile and compressive part of a fatigue cycle can be assumed. A linear fit of the low frequency cyclic test data gives a stiffness of 5.55 ± 0.05 GPa, which is very similar to the result from the quasi-static tensile test (5.84 ± 0.20 GPa). The small difference can be explained by the different machines used for the tests, including the equipment for displacement measurement. These results confirm that the novel sample design with gradient porosity presented in this study can be used for fatigue tests in compression, tension and combinations thereof.

**Figure 6:**
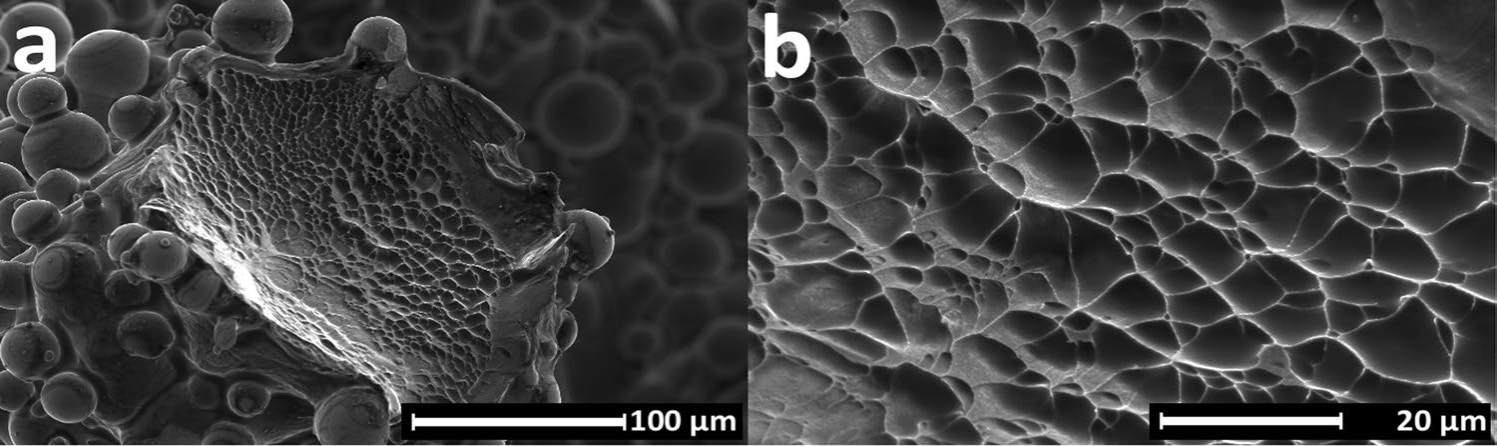
Scanning electron microscopy images of fracture surface after quasi-static tensile test: (**a**) A defect-free strut shows deformation across its entire surface. (**b**) During rupture of the struts dimples are formed.

Fractographic analysis of a sample after tensile testing showed ductile deformation and dimple formation during rupture, as can be expected for high density SLM Ti6Al4V (Fig. 6a,b)^34,35^. Fracture occurred almost always in the proximity of a node, because of the sudden change in geometry and high stresses at this location.

### Fatigue tests

**Figure 7:**
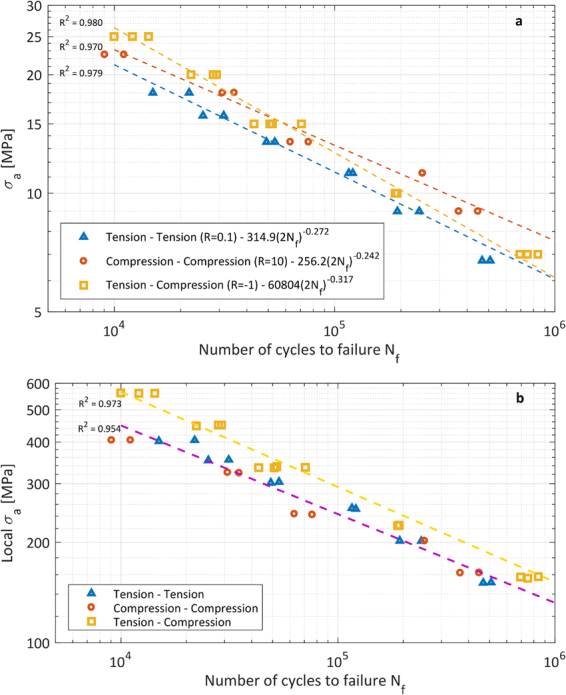
S-N curves for different load conditions: (**a**) Global stresses, on the entire cross section of the scaffold. (**b**) Maximum local tensile stresses, in a single strut.

Fatigue test results for stress ratios R = 10 (axial compression), R = 0.1 (axial tension) and R = −1 (axial tension-compression) are plotted on a double logarithmic scale and fitted by Basquin’s equation, Equation (3), in Fig. 7a. Fitting constants A and b are also given in Fig. 7a.

3 $${\sigma }_{a}=A{(2{N}_{f})}^{b}$$

Global tension-tension loading leads to a reduced fatigue life compared to global compression-compression loading. The S-N curves for both load cases are almost parallel to each other. When the same global load amplitude is applied to a scaffold, global compressive mean loads are beneficial for fatigue resistance while tensile mean loads are detrimental for fatigue life. The same effect has been reported for bulk Ti6Al4V^36^.

**Figure 8:**
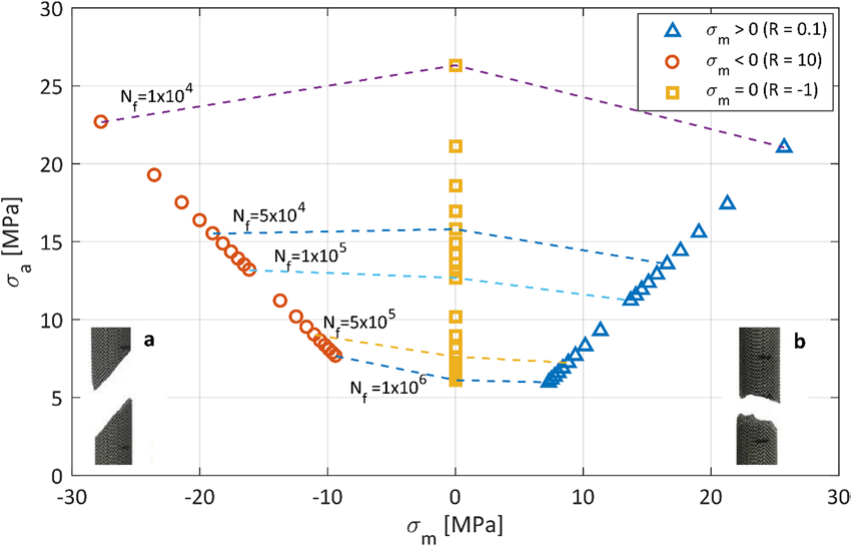
Haigh diagram for Ti6Al4V scaffolds for global stresses. Insets show the macroscopic fracture behavior for compressive fluctuating loads (**a**) and tensile fluctuating loads (**b**).

The fatigue behavior for global compression-compression (R = 10) compared to tension-compression (R = −1) is not similar to Ti6Al4V bulk material. The two S-N curves cross each other for *σ*_*a*_ = 15 MPa, which divides the data in two regions: for *σ*_*a*_ > 15 MPa the fatigue life for R = −1 is higher than for R = 10, while for *σ*_*a*_ < 15 MPa the fatigue life for R = −1 is lower than for R = 10. The Haigh diagram in Fig. 8 also shows these regions. This diagram, also known as constant-life diagram, can be used to determine the different (*σ*_*a*_, *σ*_*m*_) combinations which result in a certain amount of cycles to failure. This alternative representation of the fatigue data can be used as a predictive tool for the fatigue life of scaffolds with the addition of a mean stress. The constant cyclic life curves are plotted as dashed lines in Fig. 8 and are evaluated by fitting the three different Basquin’s equations (Fig. 7a) with constant values of the number of cycles to failure *N*_*f*_. Figure 8 can be divided in two regions, *σ*_*m*_ > 0 and *σ*_*m*_ < 0, and in each a difference in constant life curves can be observed.

For *σ*_*m*_ > 0, the constant fatigue life curves have a negative slope. This means that, in order to obtain the same fatigue resistance as the fully reversed loading condition, a lower global stress amplitude should be applied. The higher the applied mean stress, the stronger this effect. Tensile mean stresses are thus detrimental in terms of fatigue resistance. Only for a mean global tensile stress between 0 and 10 MPa and a constant fatigue life of 10^6^ cycles, there is no need to decrease the stress amplitude.

For *σ*_*m*_ < 0, three different regimes emerge: (i) for *σ*_*a*_ > 16 MPa the slope of the curve is positive, (ii) for *σ*_*a*_ between 12 and 16 MPa the curve is horizontal and (iii) for *σ*_*a*_ < 12 MPa the slope is negative. Only the last region corresponds to the behavior of bulk Ti6Al4V, where a higher stress amplitude is required to obtain the same fatigue life when the mean stress is decreased^37,38^. As highlighted by De Krijger *et al*., who studied the effect of different stress ratios on the fatigue behavior of scaffolds under compression-compression fatigue, Haigh diagrams for porous and non-porous Ti6Al4V can deviate notably^39^.

When looking at scaffolds in terms of an ensemble of struts or beams it becomes clear that global fluctuating axial loads lead to fluctuating axial and bending stresses in the struts, as shown in Fig. 2a,b. For both global compression-compression and global tension-tension, the local maximum tensile stress *σ*_1_ is a fluctuating tensile stress with R = 0.1 and high local tensile stresses occur on either the upper of lower side of a strut-node connection. For tension-compression, *σ*_1_ is a fluctuating stress with a local stress ratio R = −1 and local tensile stresses near the node occur alternately on both upper and lower sides of the strut. Based on Equations (1) and (2), an S-N curve with local stress amplitudes can be constructed, Fig. 7b.

Given the same mechanical properties of the struts and the same local stress ratio, the fatigue performance in global tension-tension and compression-compression should be the same for the same local stress amplitude. Therefore, the global tension-tension (R = 0.1) tests and the global compression-compression (R = 10) tests can be fitted by a single S-N curve in Fig. 7b. Qualitatively, Fig. 7b agrees with the behavior of bulk Ti6Al4V: global tension-tension and compression-compression loading, which both correspond to local tension-tension loading, lead to a shorter fatigue life than global tension-compression loading, which corresponds to local tension-compression loading. The reason for this is the presence of a local mean tensile stress for the first two load conditions^37,38^. As mentioned, Fig. 7b is only a qualitative interpretation of the fatigue behavior of the scaffolds since the local stress amplitude calculated here is only an approximation of the true local stresses which act on a strut. Factors which influence the local stress and are not taken into account here are: less constrained struts at the outside of the scaffold, geometrical non-linearities, stress concentrations, surface roughness, residual stresses, internal pores etc.

Figure 8 also shows the different macroscopic failure modes of the scaffolds loaded in compression-compression (a) and tension-tension fatigue (b). For compressive loads, failure occurred on an inclined plane and seemed to coincide macroscopically with the plane of maximum shear stresses. The same behavior has been observed by other researchers and has been confirmed by simulations as well^19^. In case of tensile loads, failure occurred on a plane perpendicular to the load direction.

It should be noticed that the failure modes reported in Fig. 8 are based on a sample with diamond unit cell and constant structural density. Changing the unit cell type, size, or structural density might influence the failure modes. The fracture plane for scaffolds with a cubic unit cell, for example, is oriented perpendicular to the load direction^26^. Increasing structural density would lead to fracture planes that are more and more similar to what is observed in bulk metals, while decreasing structural density might lead to fracture planes that are more and more corresponding to the direction of the unit cell struts.

**Figure 9:**
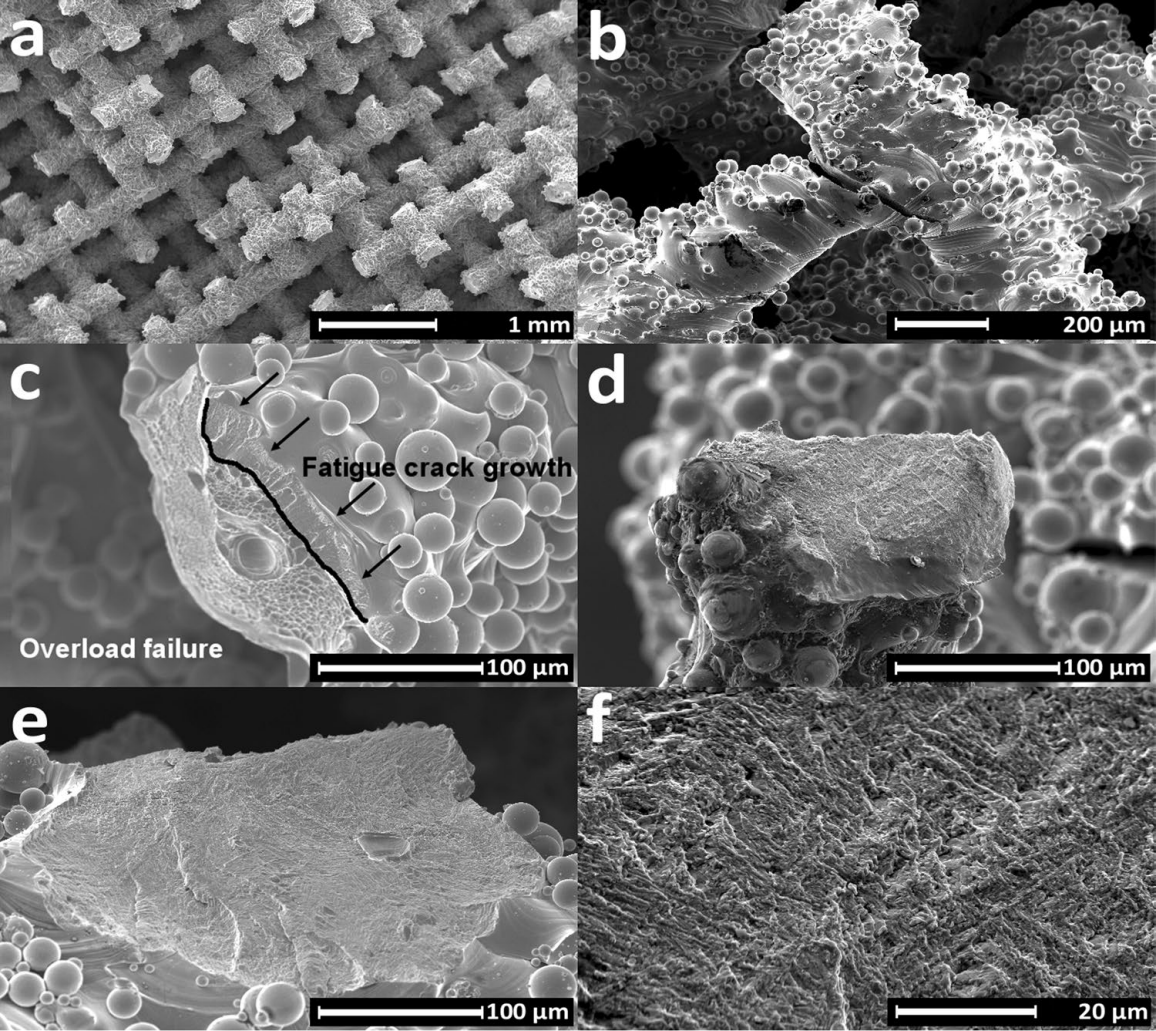
Scanning electron microscopy images of fracture surface after fatigue tests: (**a**) Samples mostly fracture close to the nodes. (**b**) A crack, which initiated and propagated close to a node for tension-compression fatigue. (**c**) The fracture surface in tension-tension fatigue showed signs of both fatigue crack propagation (upper right) and ductile overload fracture (lower left). (**d**,**e**) The fracture surface after compression-compression fatigue failure (**d**) and tension-compression fatigue failure (**e**) showed only signs of fatigue crack propagation, no overload failure. (**f**) Higher magnification of crack propagation area.

Independent of the stress ratio and the shape of the macroscopic fracture surface, the struts fractured close to the nodes, Fig. 9a. It is clear from Fig. 2 that this is a location with maximum stress and thus accelerated crack initiation and propagation, Fig. 9b. The same failure location was found in tension-compression fatigue of Ti6Al4V scaffolds with cubic unit cells^26^. After tension-tension fatigue testing, the fracture surface consisted of a crack propagation region and overload region with dimples, Fig. 9c. For this strut, the crack started at the upper right corner and progressed towards the lower left until a sudden and complete failure of the strut. Pores were observed in a minority of the struts during fractographic analysis. The presence of the pores weakens the struts but no sign of crack initiation at a pore was found. This is consistent with the results obtained for tension-compression fatigue tests on scaffolds with a cubic unit cell and shows that more work to reduce the surface roughness of SLM scaffolds is required^26^. No clear overload zone with dimples was observed on fracture surfaces after tension-compression and compression-compression fatigue, Fig. 9d,e. The appearance of the crack propagation region in individual struts is similar to what has been described for bulk SLM Ti6Al4V and consists of small irregular facets and secondary cracks caused by a brittle crack propagation mechanism, Fig. 9f ^40,41^.

## Conclusion

The novel sample design with a porosity gradient between the solid ends and scaffold middle presented in this study was successfully used to perform quasi-static tension, tension-tension (R = 0.1), tension-compression (R = −1) and compression-compression (R = 10) fatigue tests. The Haigh diagram shows that, for the R-values tested here, a decrease of global stress amplitude is necessary if a mean global tensile stress is applied and a constant fatigue life is required. For a small mean global compressive stress, up to 12 MPa, an increase in global stress amplitude is required to maintain a constant fatigue life. For a higher mean global compressive stress, a decrease of global stress amplitude is necessary if a constant fatigue life is required. When fatigue life is interpreted in terms of local stresses, the behavior of single struts is shown to be qualitatively the same as bulk Ti6Al4V. Compression-compression and tension-tension fatigue regimes lead to a shorter fatigue life than fully reversed loading due to the presence of a local mean tensile stress. Fractographic analysis showed that most fracture sites were located close to the nodes, where the highest tensile stresses are located.

## References

[1] Geetha M, Singh AK, Asokamani R, Gogia AK (2009). Ti based biomaterials, the ultimate choice for orthopaedic implants – A review. Prog. Mater. Sci..

[2] Herzog D, Seyda V, Wycisk E, Emmelmann C (2016). Additive manufacturing of metals. Acta Mater..

[3] Li P, Warner DH, Fatemi A, Phan N (2016). Critical assessment of the fatigue performance of additively manufactured Ti–6Al–4V and perspective for future research. Int. J. Fatigue.

[4] Tan XP, Tan YJ, Chow CSL, Tor SB, Yeong WY (2017). Metallic powder-bed based 3D printing of cellular scaffolds for orthopaedic implants: A state-of-the-art review on manufacturing, topological design, mechanical properties and biocompatibility. Mater. Sci. Eng. C.

[5] van der Stok J (2015). Full regeneration of segmental bone defects using porous titanium implants loaded with BMP-2 containing fibrin gels. Eur. Cell. Mater..

[6] Li, G. *et al*. *In vitro* and *in vivo* study of additive manufactured porous Ti6Al4V scaffolds for repairing bone defects. *Sci. Rep*. **6** (2016). 10.1038/srep34072 PMC5036184 27667204

[7] Zadpoor AA (2017). Mechanics of additively manufactured biomaterials. J. Mech. Behav. Biomed. Mater..

[8] Wycisk, E., Emmelmann, C., Siddique, S. & Walther, F. High Cycle Fatigue Performance of Ti-6Al-4V processed by Selective Laser Melting. **816–817**, 134–139 (2013).

[9] Khairallah SA, Anderson AT, Rubenchik A, King WE (2016). Laser powder-bed fusion additive manufacturing: Physics of complex melt flow and formation mechanisms of pores, spatter, and denudation zones. Acta Mater..

[10] Cheng, B. & Chou, K. Melt Pool Evolution Study in Selective Laser Melting. in *Proceedings of the 26th Annual International Solid Freeform Fabrication Symposium* (2015).

[11] Zhao, C. *et al*. Real-time monitoring of laser powder bed fusion process using high-speed X-ray imaging and diffraction. *Sci. Rep*. **7** (2017). 10.1038/s41598-017-03761-2 PMC5472560 28620232

[12] Xu Z, Wen W, Zhai T (2012). Effects of Pore Position in Depth on Stress/Strain Concentration and Fatigue Crack Initiation. Metall. Mater. Trans. A.

[13] Tammas-Williams S, Withers PJ, Todd I, Prangnell PB (2017). The Influence of Porosity on Fatigue Crack Initiation in Additively Manufactured Titanium Components. Sci. Rep..

[14] Bagheri ZS, Melancon D, Liu L, Johnston RB, Pasini D (2017). Compensation strategy to reduce geometry and mechanics mismatches in porous biomaterials built with Selective Laser Melting. J. Mech. Behav. Biomed. Mater..

[15] Hrabe NW, Heinl P, Flinn B, Körner C, Bordia RK (2011). Compression-compression fatigue of selective electron beam melted cellular titanium (Ti-6Al-4V). J. Biomed. Mater. Res. B Appl. Biomater..

[16] Li SJ (2012). Compression fatigue behavior of Ti–6Al–4V mesh arrays fabricated by electron beam melting. Acta Mater..

[17] Amin Yavari S (2013). Fatigue behavior of porous biomaterials manufactured using selective laser melting. Mater. Sci. Eng. C.

[18] Amin Yavari S (2015). Relationship between unit cell type and porosity and the fatigue behavior of selective laser melted meta-biomaterials. J. Mech. Behav. Biomed. Mater..

[19] Zargarian A, Esfahanian M, Kadkhodapour J, Ziaei-Rad S (2016). Numerical simulation of the fatigue behavior of additive manufactured titanium porous lattice structures. Mater. Sci. Eng. C.

[20] Zhao S (2016). The influence of cell morphology on the compressive fatigue behavior of Ti-6Al-4V meshes fabricated by electron beam melting. J. Mech. Behav. Biomed. Mater..

[21] Hedayati R, Hosseini-Toudeshky H, Sadighi M, Mohammadi-Aghdam M, Zadpoor AA (2016). Computational prediction of the fatigue behavior of additively manufactured porous metallic biomaterials. Int. J. Fatigue.

[22] Li F, Li J, Huang T, Kou H, Zhou L (2017). Compression fatigue behavior and failure mechanism of porous titanium for biomedical applications. J. Mech. Behav. Biomed. Mater..

[23] Van Hooreweder B, Apers Y, Lietaert K, Kruth J-P (2017). Improving the fatigue performance of porous metallic biomaterials produced by Selective Laser Melting. Acta Biomater..

[24] Bobbert FSL (2017). Additively manufactured metallic porous biomaterials based on minimal surfaces: A unique combination of topological, mechanical, and mass transport properties. Acta Biomater..

[25] Brenne F, Niendorf T, Maier HJ (2013). Additively manufactured cellular structures: Impact of microstructure and local strains on the monotonic and cyclic behavior under uniaxial and bending load. J. Mater. Process. Technol..

[26] Dallago M (2018). Fatigue and biological properties of Ti-6Al-4V ELI cellular structures with variously arranged cubic cells made by selective laser melting. J. Mech. Behav. Biomed. Mater..

[27] ISO. ISO 13314 - Mechanical testing of metals - Ductility testing - Compression test for porous and cellular metals (2011).

[28] ASTM International. ASTM Standard F3001, 2014, ‘Standard Specification for Additive Manufacturing Titanium-6Aluminium-4Vanadium ELI (Extra Low Interstitial) with Powder Bed Fusion’ (2014).

[29] Ahmadi SM (2014). Mechanical behavior of regular open-cell porous biomaterials made of diamond lattice unit cells. J. Mech. Behav. Biomed. Mater..

[30] Dallago M (2017). Fatigue properties of Ti6Al4V cellular specimens fabricated via SLM: CAD vs real geometry. Struct. Integr. Procedia.

[31] Onck PR, Andrews EW, Gibson LJ (2001). Size effects in ductile cellular solids. Part I: modeling. Int. J. Mech. Sci..

[32] Andrews EW, Gioux G, Onck P, Gibson LJ (2001). Size effects in ductile cellular solids. Part II: experimental results. Int. J. Mech. Sci..

[33] Kadkhodapour J (2015). Failure mechanisms of additively manufactured porous biomaterials: Effects of porosity and type of unit cell. J. Mech. Behav. Biomed. Mater..

[34] Rafi HK, Starr TL, Stucker BE (2013). A comparison of the tensile, fatigue, and fracture behavior of Ti–6Al–4V and 15-5 PH stainless steel parts made by selective laser melting. Int. J. Adv. Manuf. Technol..

[35] Gong H (2015). Influence of defects on mechanical properties of Ti–6Al–4V components produced by selective laser melting and electron beam melting. Mater. Des..

[36] Dowling, N. E. Estimating Fatigue Life. in *Fatigue and Fracture* **19**, 250–262 (ASM International, 1996).

[37] Prevéy PS, Jayaraman N, Ravindranath RA, Shepard M (2008). Improved High Cycle Fatigue Damage Tolerance of Turbine-Engine Compressor Components by Low Plasticity Burnishing. J. Eng. Gas Turbines Power.

[38] Benedetti M, Fontanari V, Bandini M, Zanini F, Carmignato S (2018). Low- and high-cycle fatigue resistance of Ti-6Al-4V ELI additively manufactured via selective laser melting: Mean stress and defect sensitivity. Int. J. Fatigue.

[39] de Krijger J (2017). Effects of applied stress ratio on the fatigue behavior of additively manufactured porous biomaterials under compressive loading. J. Mech. Behav. Biomed. Mater..

[40] Van Hooreweder B, Moens D, Boonen R, Kruth J-P, Sas P (2012). Analysis of Fracture Toughness and Crack Propagation of Ti6Al4V Produced by Selective Laser Melting. Adv. Eng. Mater..

[41] Konečná R, Kunz L, Bača A, Nicoletto G (2017). Resistance of direct metal laser sintered Ti6Al4V alloy against growth of fatigue cracks. Eng. Fract. Mech..

